# Double Subclavian Steal Syndrome As Initial Manifestation of Erdheim-Chester Disease: A Case Report

**DOI:** 10.7759/cureus.50427

**Published:** 2023-12-13

**Authors:** Josef Finsterer

**Affiliations:** 1 Neurology, Neurology and Neurophysiology Center, Vienna, AUT

**Keywords:** aortal sheathing, neuropathy, histiocytosis, erdheim-chester disease, subclavian steal

## Abstract

Erdheim-Chester disease (ECD) is a histiocytosis that infiltrates all organs, but especially large arteries such as the aorta and its branches, which become sheathed and lead to multiple stenoses or even occlusions. Subclavian steal syndrome (SSS) has not been reported in ECD. A 68-year-old female was diagnosed with ECD due to the typical sheathing of the aorta and its major branches. Five years previously, asymptomatic SSS due to stenosis of the left subclavian artery was incidentally diagnosed. In the following years, occlusion of the subclavian and left vertebral artery, and stenosis of basilar artery and right middle cerebral artery occurred. The abnormal cerebral perfusion had consequences on the perfusion of the left upper extremity and was presumably responsible for falls. Basilar and middle cerebral artery stenosis is rare in ECD and vertebral artery occlusion and double subclavian steal have not been reported in ECD. This case is the first to show that the initial manifestation of ECD can be unilateral SSS and that subclavian artery occlusion can even lead to double SSS. Patients with SSS should undergo a thorough diagnostic evaluation to detect rare causes of SSS, such as ECD.

## Introduction

Erdheim-Chester disease (ECD), also known as non-Langerhans-cell histiocytosis, is a histiocytic neoplasm, like Langerhans cell histiocytosis and Rosai Dorfman disease, characterized by a multisystem disease including bones, heart, lungs, arteries and central nervous system (CNS) and less commonly the kidneys, eyes, retroperitoneum, and skin [1,2]. The most common clinical manifestations are bone pain, mostly in the lower extremities, and diabetes insipidus [1]. Diagnostic workup typically reveals circumferential sheathing of the entire aorta with soft tissue containing abnormal histiocytes (coated aorta) and its main branches, cerebral disease, and long bone infiltration [3]. Circumferential sheathing of the aorta can be complicated by stenosis of multiple arteries [4,5] and even by thrombosis or occlusion of affected arterial segments [3]. Subclavian artery occlusion and the resulting double subclavian steal syndrome (SSS) have not been reported as a complication of ECD and particularly not as an initial manifestation of the disease.

## Case presentation

**Figure 1:**
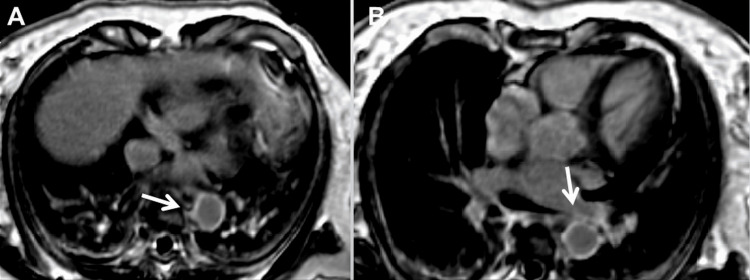
Computed tomography of the thorax showing “coating” of the thoracic aorta (A, B), suggestive of ECD.

**Figure 2:**
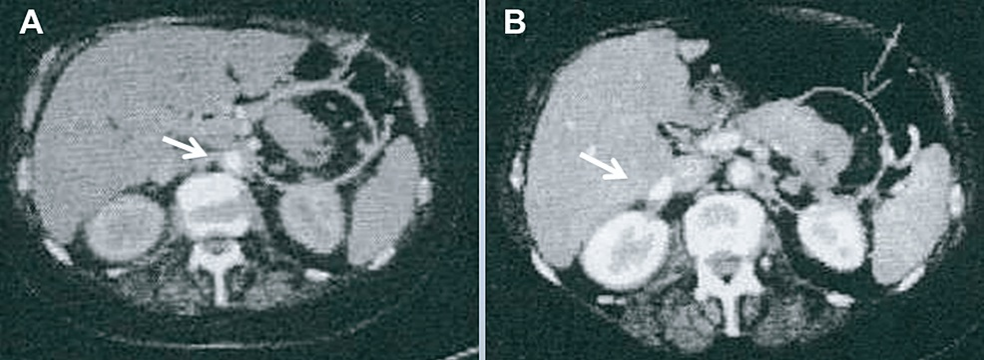
Positron emission tomography (PET)-CT (A) and CT with contrast medium of the abdomen showing enhancement of the corpus and cauda (B) of the pancreas, suggestive of pancreatitis.

The patient is a 68-year-old female with ECD, first diagnosed one month before admission due to typical coating (up to 4.6mm) of the aorta (Figures 1A, 1B), atypical CD68-positive histiocytes, myocarditis, pericarditis with pericardial effusion, perinephritis (“hairy kidney”), multiple arterial stenoses, and continuously elevated C-reactive protein. The patient also had pancreatitis (Figures 2A, 2B). There was no evidence of pituitary impairment (diabetes insipidus), bone involvement, or lung involvement. She has not yet been tested for BRAF (V600E), NRAS, PIK3CA, MAP2K1, KRAS, ARAF, or IDH2 variants. ECD was initially treated with steroids and tocilizumab (8 mg/kg), which had limited beneficial effects, so she was switched to interferon-alpha (9 mIU/week). At the age of 63 years, unilateral SSS was first incidentally diagnosed. Initially, the subclavian artery was judged to be severely stenotic, but on follow-up examination, occlusion occurred. Severe stenosis of the celiac trunk, superior mesenteric artery, and renal arteries were later discovered.

**Figure 3:**
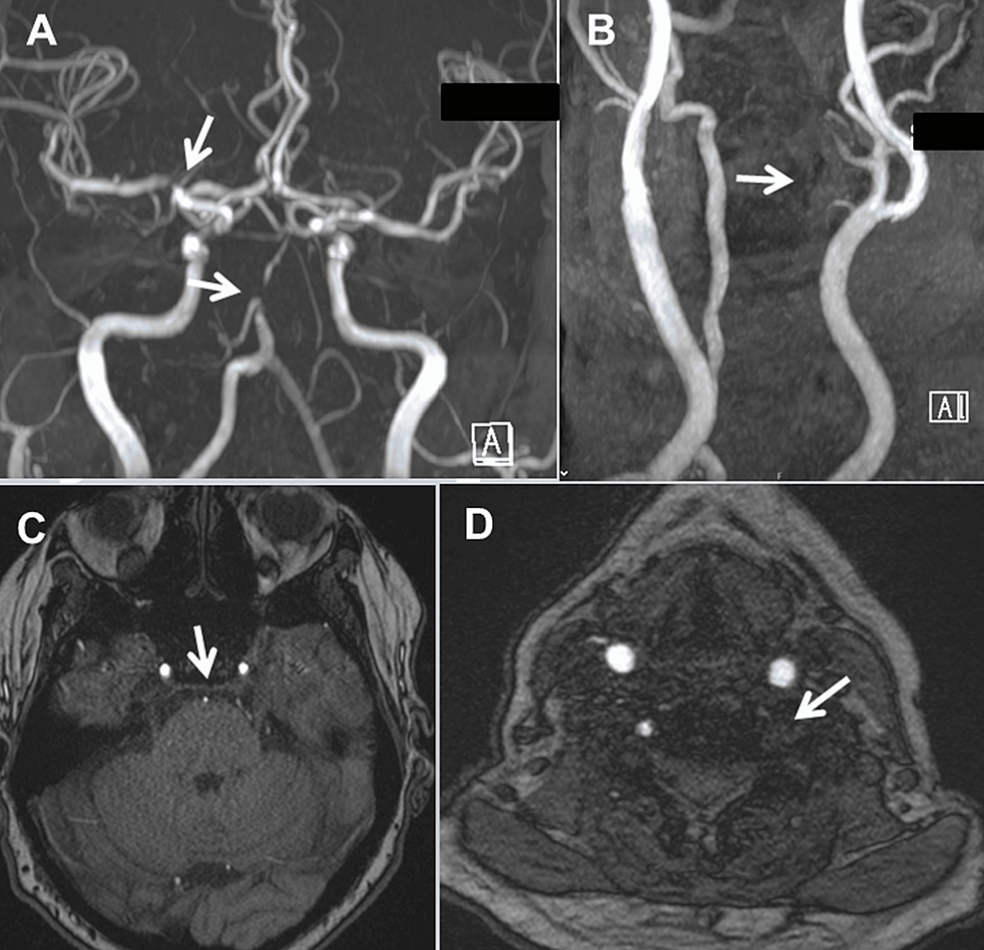
Magnetic resonance angiography showing filiform stenosis of the basilar artery and stenosis of the right middle cerebral artery (A, C). The left vertebral artery is only moderately contrasted (A). The proximal segments of the left vertebral artery are occluded (B, D).

At age 68, severe stenosis of the basilar cerebral artery, right middle cerebral artery, and occlusion of the left vertebral artery were also noted (Figures 3A-3D). The splenic artery and vein were also occluded. Medical history was also positive for adolescent migraine, left-sided glaucoma, Helicobacter-positive gastritis, hyponatremia, protein-C elevation, osteopenia, sigmoid colon diverticulosis, arterial hypertension, which was well controlled with antihypertensives at home and during hospital stays, hyperlipidemia, which was also well controlled (low-density lipoprotein <70 mg/dL), and vitamin-D deficiency.

**Figure 4:**
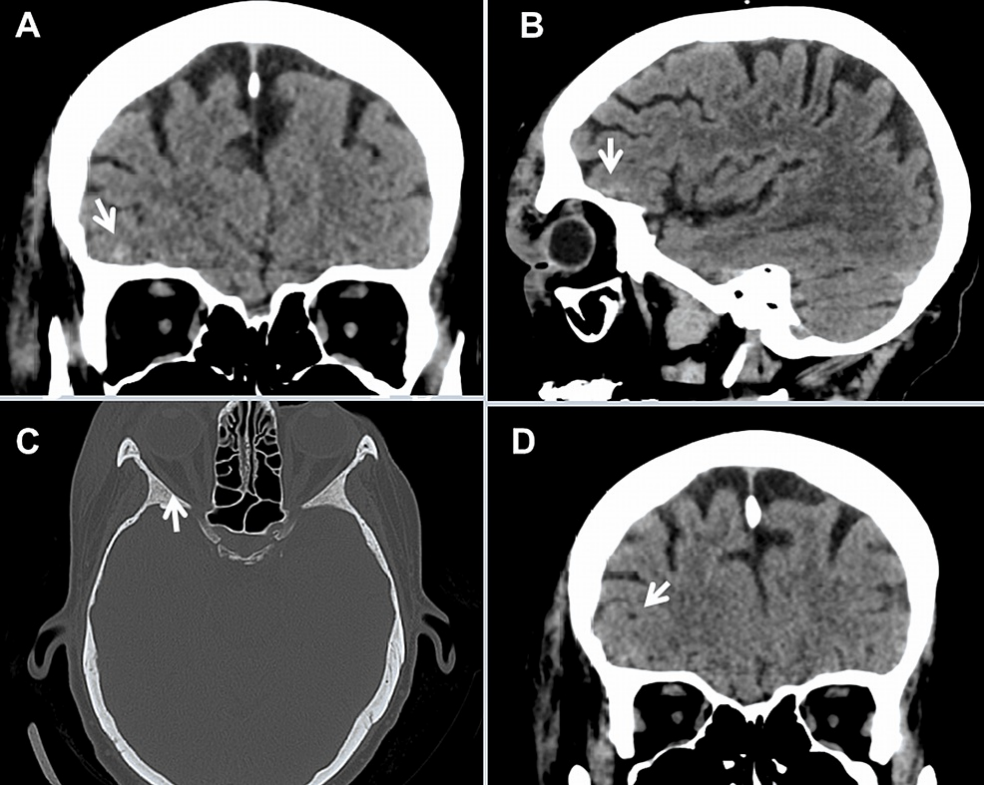
Cerebral CT scan showing hyperdensity in the right fronto-polar region (coronary view (A, D), sagittal view (B), and bone window (C)) being interpreted as subarachnoid bleeding. Since computed tomography angiography did not disclose an aneurysm and since the history was positive for a fall, the lesion was interpreted as traumatic subarachnoid bleeding.

The patient was most recently admitted following a fall complicated by a small, presumably traumatic subarachnoid bleeding that resolved without intervention (Figures 4A-4D). The clinical neurological examination revealed mild (M5-), diffuse (proximal and distal) quadriparesis with left-sided predominance without sensory disturbances, generally decreased tendon reflexes, and rapid leg muscle fatigue after 10 seconds in the leg extension test. Blood tests revealed mild anemia, moderate renal insufficiency, elevated erythrocyte sedimentation rate, mild C-reactive protein elevation, mild leukocytosis, hyperglycemia, and HbA1c of 7.1. IgG4 was normal as were ANA, DFS70, antiphospholipid antibodies (APLAs), lupus coagulant, and a screening for thrombophilia. Doppler ultrasound examination of the subclavian, ulnar, and radial arteries showed a biphasic signal on the left side but a normal triphasic signal on the right side. Upper extremity oscillometry showed normal amplitude on the right side but decreased amplitude on the left side. Magnetic resonance angiography (MRA) of the extra- and intra-cranial arteries showed filling of the left internal and common carotid arteries and occlusion of the left vertebral artery (Figure 3), but reversal of blood flow in the left internal carotid artery on ultrasound. Abdominal computed tomography (CT) showed hydronephrosis I, pancreatitis, and a mass lesion in the corpus-cauda transition zone of the pancreas (Figure 2), which was identified on biopsy as chondroid reactive inflammatory tissue. There was a thrombosis of both the splenic artery and the splenic vein, which was complicated by a pronounced fundic varicosis. Echocardiography showed mild pericardial effusion and mild left ventricular hypertrophy. Cardiac MRI showed normal systolic function but mild late enhancement in the basal, middle, and anterolateral myocardium and a 15-mm pericardial effusion.

Current medications included methyl-prednisolone, interferon-alpha, candesartan, amlodipine, atorvastatin, metformin, acetyl-salicylic acid, calcium, moxonidine, bimatoprost, borzolamide, vitamin-D, and pantoprazole.

## Discussion

The patient is interested in SSS as the first manifestation of ECD because high-grade stenosis and subsequent occlusion of the left subclavian artery with consecutive SSS were not reported in ECD. However, arterial stenosis or even arterial occlusions are common in ECD and have previously been reported as a complication of ECD [1,4,5]. For example, in a 76-year-old male with ECD manifesting as heart failure and pericardial effusion, a typical coating of the aorta was described that began at the aortic valve and ended at the inferior mesenteric artery [4]. It resulted in stenosis of the left subclavian, celiac trunk, renal, and upper mesenteric arteries [4]. Stenosis of the renal and superior mesenteric artery was treated with angioplasty and stenting [4]. Several other cases of stenosis or occlusion of large arteries can be found in the literature. Even basilar artery stenosis has previously been described as a complication of ECD [6].

In general, SSS is multifactorial and has been associated with Takayasu arteritis, thoracic outlet syndrome due to cervical rib, neuromuscular compression, overexertion (cricket bowlers, baseball pitchers), after surgical repair of coarctation, congenital right aortic arch, aortic dissection, vertebral artery congenital malformations, or external vertebral artery compression [7]. Subclavian stenosis is asymptomatic in most patients, but interarm blood pressure differences, arm pain, fatigue, numbness, or paresthesias have been reported [6]. There are also reports of SSS clinically manifesting with dizziness, blurred vision, vertigo, disequilibrium, ataxia, tinnitus, drop attacks, or syncope, especially after vigorous exercise of the left arm. Carotid ultrasound may reveal carotid artery occlusion or vertebral artery disease. The index patient’s drop attack could be due to this pathophysiological mechanism. In general, double SSS has been rarely reported [8], but not in a patient with ECD. Regarding the index patient’s SSS, it was suspected that the left arm received blood only via the left carotid artery because the left vertebral artery and the left subclavian artery were occluded. Flow reversal in the left carotid artery has been previously reported in cases with double SSS [8,9], but not in a patient with ECD.

The mild quadriparesis in the index patient was explained by neuropathy because there was no evidence of myopathy and CNS causes of quadriparesis were largely ruled out. A strong argument against quadriparesis due to CNS disease is that tendon reflexes are generally reduced. However, the cause of neuropathy remains speculative as diabetes was newly discovered and therefore cannot be blamed for the neuropathy. There was also no kidney failure and no history of alcoholism or chemotherapy. However, neuropathy could be a manifestation of ECD, as neuropathy has occasionally been reported as a manifestation of ECD.

Perinephritis was considered a manifestation of ECD because renal involvement is common in ECD and because hairy kidneys, perinephric masses, perinephritis, and perinephric calcification in ECD have been repeatedly reported [10]. Whether the slight myocardial enhancement during contrast agent administration actually represents myocarditis or rather myocardial fibrosis, remains unclear because no myocardial biopsy was performed. However, myocardial fibrosis has recently been reported as a complication of ECD [11]. Glaucoma was not regarded as a manifestation of ECD since it has not previously been reported in association with ECD. In general, ocular manifestations of ECD are rare and include orbital masses, orbital infiltration, choroidal infiltration, eyelid swelling, periocular xanthoma, and nystagmus [12,13].

**Table 1:** Clinical manifestations of ECD in the index case and in patients previously reported.

Manifestation	Index case	Literature	Reference
SSS	yes	no	none
Celiac trunk stenosis	yes	yes	[4]
Diabetes	yes	yes	[14]
Superior mesenteric artery stenosis	yes	yes	[15]
Renal artery stenosis	yes	yes	[16]
Hyperlipidemia	yes	yes	[17]
Pancreatitis	yes	yes	[18]
Diverticulosis	yes	no	none
Subarachnoid bleeding	yes	no	none
Osteopenia	yes	yes	[19]
Migraine	yes	yes	[20]
Splenic artery/vein thrombosis	yes	no	none

## Conclusions

This case is the first to show that the initial manifestation of ECD can be SSS and one of the few cases to show that ECD can also manifest with cerebral artery stenosis or occlusions. As the disease progresses, occlusion of the subclavian artery and vertebral artery may occur, resulting in double SSS. The complex pathological blood flow can be responsible for circulatory deficits in the left arm and falls. Patients with SSS should undergo a thorough diagnostic evaluation to detect rare causes of SSS, such as ECD. In ECD cases, there is also a need to monitor cerebral blood flow regularly. Extensive research is required to develop the most appropriate therapeutic strategies for intra- and extracranial artery stenosis or occlusion in ECD.

## References

[1] Benson JC, Vaubel R, Ebne BA (2023). Erdheim-Chester disease. AJNR Am J Neuroradiol.

[2] Goyal G, Young JR, Koster MJ (2019). The Mayo Clinic histiocytosis working group consensus statement for the diagnosis and evaluation of adult patients with histiocytic neoplasms: Erdheim-Chester disease, Langerhans cell histiocytosis, and Rosai-Dorfman disease. Mayo Clin Proc.

[3] He J, Fang X, Zhang X, Chen K, Huang J (2022). Extensive aortic thromboembolism in a patient with Erdheim-Chester disease: a case report. Front Cardiovasc Med.

[4] Vega J, Cisternas M, Bergoeing M, Espinosa R, Zapico A, Chadid P, Santamarina M (2011). Erdhei-Chester disease: report of one case (Article in Spanish). Rev Med Chil.

[5] Lakhani PM, Borysiewicz C, Mason J (2022). Erdheim-Chester disease: a rare cause of bilateral renal artery stenosis, mimicking large vessel vasculitis. BMJ Case Rep.

[6] Mathis S, Godenèche G, Haroche J (2016). Long-term outcome of basilar stenosis in Erdheim-Chester disease: a case report. Medicine (Baltimore).

[7] Shankar Kikkeri N, Nagalli S (2023). Subclavian Steal Syndrome.

[8] Rizza A, De Caterina AR, Murzi M, Farneti PA, Palmieri C, Berti S (2019). Double carotid-subclavian bypass followed by endovascular exclusion of a Kommerell diverticulum and bilateral subclavian artery occlusion in a right-sided aortic arch. JACC Cardiovasc Interv.

[9] Leach DF 3rd, Radwanski DM, Kaur P, Das DD, Kondapalli M (2023). Recurrent subclavian steal syndrome: a novel case of vasculopathy. Cureus.

[10] Singh R, Naranje P, Ramateke P, Damle NA (2021). Erdheim-Chester disease: an unusual aetiology of bilateral lipomatous perinephric masses. BMJ Case Rep.

[11] Palmisano A, Campochiaro C, Vignale D (2023). Cardiovascular involvement in Erdheim-Chester diseases is associated with myocardial fibrosis and atrial dysfunction. Radiol Med.

[12] Alzahem T, Alkatan HM, Maktabi AM, Alsulaiman N, Cruz AA (2023). Ophthalmic histiocytic lesions (diseases of the L group): a multicenter clinicopathological study of 18 cases and review of literature. Eur J Ophthalmol.

[13] Qiao J, Ma R, Peng X, He W (2023). Erdheim-Chester disease with bilateral orbital masses and multi-systemic symptoms: two case reports. World J Surg Oncol.

[14] Chen M, Ding C, Lu T, Niu N, Han B (2018). Langerhans cell histiocytosis and Erdheim-Chester disease overlap syndrome with bone marrow involvement and type 2 diabetes mellitus. Ann Hematol.

[15] Wang F, Cao X, Niu N, Zhang Y, Wang Y, Feng F, Jin Z (2019). Multisystemic imaging findings in Chinese patients with Erdheim-Chester disease. AJR Am J Roentgenol.

[16] Nikpanah M, Kim L, Mirmomen SM (2018). Abdominal involvement in Erdheim-Chester disease (ECD): MRI and CT imaging findings and their association with BRAF(V600E) mutation. Eur Radiol.

[17] Akin EA, Osman M, Ellenbogen AL (2018). FDG PET/CT findings of Erdheim-Chester disease: radiologic response to a novel treatment regimen. Clin Nucl Med.

[18] Rafiee MJ, Taylor J, Hickeson M, Friedrich MG, Chetrit M (2023). Pancreatic involvement in Erdheim-Chester disease: rare presentation of a rare disease. Radiol Case Rep.

[19] Saini R, DiFrancesco LM, Johnston K, Khan A, Kline GA (2019). Diffuse, fracturing systemic skeletal histiocytosis of unknown type: a novel metabolic bone disease. Osteoporos Int.

[20] Johnson MD, Aulino JP, Jagasia M, Mawn LA (2004). Erdheim-chester disease mimicking multiple meningiomas syndrome. Am J Neuroradiol.

